# Integrated multi-omics and single-cell analysis identify SERPINE1 as a key mediator of the inflammatory tumor microenvironment in PDAC

**DOI:** 10.3389/fimmu.2025.1716878

**Published:** 2026-01-12

**Authors:** Di Wang, Qing Chen, Can-ming Li, Yan Xie, Chunhui Yuan, Ren Lang, Wen-Tao Jiang

**Affiliations:** 1 Department of Liver Transplantation, First Central Hospital of Tianjin Medical University, Tianjin, China; 2 Tianjin Key Laboratory of Molecular and Treatment of Liver Cancer, Tianjin First Central Hospital, Tianjin, China; 3 Department of General Surgery, Peking University Third Hospital, Beijing, China; 4 Department of General Surgery, Tianjin Fifth Central Hospital, Tianjin, China; 5 Department of Hepatobiliary and Pancreaticosplenic Surgery, Beijing Chaoyang Hospital, Capital Medical University, Beijing, China

**Keywords:** inflammatory tumor microenvironment, multi-omics, pancreatic ductal adenocarcinoma, SERPINE1, single-cell RNA sequencing

## Abstract

**Background:**

Chronic inflammation is increasingly recognized as a fundamental driver of pancreatic ductal adenocarcinoma (PDAC) initiation and progression. Although numerous bioinformatics studies have characterized genetic alterations in PDAC, the key inflammatory regulators that bridge tumor cells and the immunosuppressive stroma remain unclear.

**Methods:**

We conducted an integrative multi-omics analysis of TCGA, GEO, and ArrayExpress datasets to define inflammation-associated molecular signatures in PDAC. Differentially expressed genes were analyzed through pathway enrichment, protein–protein interaction modeling, and immune infiltration profiling. Immunotherapeutic relevance was assessed using the IMvigor210 cohort and TIDE algorithm, while drug repurposing candidates were identified via molecular docking. Single-cell RNA sequencing and *in vitro* functional assays were employed to validate gene expression patterns and mechanistic functions within the PDAC microenvironment.

**Results:**

Our multi-cohort analysis revealed a robust inflammation-associated gene network in PDAC, with SERPINE1 emerging as a consistent central hub. Elevated *SERPINE1* expression was tightly linked to a profoundly immunosuppressive tumor microenvironment and predicted diminished responsiveness to immunotherapy across datasets. Structure-based molecular docking further identified Lenvatinib and Dasatinib as previously unappreciated candidate inhibitors of SERPINE1, suggesting actionable therapeutic opportunities. Single-cell transcriptomic profiling resolved nine major cellular compartments and pinpointed fibroblasts as the principal stromal niche orchestrating SERPINE1-driven crosstalk between inflammation and immune evasion, a cellular origin that has not been systematically defined before. Translational analyses demonstrated consistently elevated SERPINE1 in tumor tissues, and functional validation using CRISPR-mediated knockout in PDAC cell lines significantly impaired proliferation and migration while inducing robust apoptosis, thereby establishing SERPINE1 as a previously underappreciated but essential driver of PDAC aggressiveness.

**Conclusions:**

This integrative multi-omics and single-cell analysis establishes SERPINE1 as a central orchestrator of inflammation-driven stromal remodeling and immune evasion in PDAC. Its strong prognostic power, combined with newly revealed druggability, positions SERPINE1 as a tractable therapeutic axis for precision immunotherapy and rational drug repurposing. These findings provide a mechanistically grounded and clinically actionable entry point into targeting the inflammatory tumor microenvironment of pancreatic cancer.

## Introduction

Pancreatic ductal adenocarcinoma (PDAC) remains one of the most lethal malignancies worldwide, characterized by late clinical presentation, rapid metastatic dissemination, and profound resistance to current therapies (1). Despite substantial progress in genomic and transcriptomic profiling, the molecular determinants that drive PDAC progression and underlie its highly aggressive phenotype remain only partially understood. Over the last decade, numerous bioinformatics studies using TCGA, GEO, and other large-scale transcriptomic datasets have sought to delineate genetic programs associated with PDAC initiation, progression, and treatment response. These analyses have highlighted dysregulated pathways involving mutational networks (2), inflammatory signaling (3), extracellular matrix (ECM) remodeling (4), and immune evasion (5), and have proposed a wide range of putative biomarkers for prognostication and molecular stratification. Large-scale integrative studies have also reaffirmed recurrent genetic alterations—including KRAS, TP53, SMAD4, and CDKN2A—as central drivers of PDAC evolution and hallmarks of its molecular landscape (6). However, these canonical pathways alone do not fully account for the extensive intertumoral heterogeneity or the complex stromal and immune microenvironment that shapes PDAC behavior. Accordingly, comprehensive multi-omics approaches that couple tumor-intrinsic alterations with microenvironmental remodeling are urgently needed to reveal novel mechanisms of disease progression and uncover actionable therapeutic vulnerabilities.

The inflammatory milieu of the pancreas is widely recognized as a key driver of tumor initiation and progression, particularly in the setting of chronic pancreatitis (CP) (7). Within this context, activated immune cells release abundant cytokines and chemokines that not only facilitate tumor cell migration but also impair the immune system’s ability to detect and eliminate malignant cells (8). Among the inflammatory mediators shaping this tumor‐promoting microenvironment, serine protease inhibitor clade E member 1 (SERPINE1), also known as plasminogen activator inhibitor-1 (PAI-1), has emerged as a multifaceted regulator of cancer biology. Historically, SERPINE1 has been primarily studied in the context of thrombosis. However, accumulating evidence now demonstrates that SERPINE1 is frequently overexpressed across diverse cancers and chronic inflammatory conditions. Functionally, SERPINE1 regulates extracellular matrix (ECM) remodeling, angiogenesis, and immune evasion by inhibiting tissue-type and urokinase-type plasminogen activators. Recent studies further highlight its tumor-promoting functions, showing that SERPINE1 enhances progression and metastasis in breast, gastric, and head and neck cancers (9–11). In breast cancer, high SERPINE1 expression correlates with poor prognosis, increased cellular motility, proliferative capacity, and chemoresistance (12). Moreover, pharmacologic inhibition of SERPINE1 has been shown to suppress glioma metastasis through blockade of the PI3K/AKT signaling pathway (13). A pan-cancer analysis further showed that SERPINE1 is broadly upregulated across multiple tumor types, and that its elevated expression is consistently associated with poor prognosis and increased metastatic potential (14). However, a comprehensive characterization of its role in PDAC is still lacking. Beyond its effects on tumor cells themselves, the mechanistic basis of its activity within the PDAC tumor microenvironment—particularly its interactions with stromal and immune components—remains unclear, underscoring the need for deeper investigation.

To address these knowledge gaps, we conducted an integrative analysis across TCGA, GEO, and ArrayExpress cohorts to identify inflammation-associated genes in PDAC and to define their prognostic and immunological relevance. In this study, we employed comprehensive multi-omics approaches together with single-cell RNA sequencing to characterize the role of SERPINE1 within the PDAC tumor microenvironment. Among candidates, SERPINE1 consistently emerged as an upregulated gene with strong prognostic significance. By surveying multiple public cancer datasets, we also systematically evaluated SERPINE1 expression patterns across malignancies and its associations with clinical outcomes, immune infiltration, therapeutic response, and key molecular features including transcriptomic signatures, DNA methylation, and somatic mutations. Collectively, these analyses identified SERPINE1 as a central stromal-associated hub gene, closely related to matrix activation and adverse clinical outcomes. We further integrated bulk transcriptomic profiling with single-cell datasets to uncover a previously unrecognized link between SERPINE1 expression, specific CAF subsets, immunosuppressive stromal features, and may contribute to extracellular matrix remodeling and an immunosuppressive microenvironment. Our findings not only reinforce SERPINE1 as a critical driver of PDAC progression but also highlight its potential as a therapeutic target positioned at the interface between tumor and stroma.

## Materials and methods

### Data collecting

We collected the gene transcriptome data and clinical information of 178 PDAC patients from the TCGA database. Patients with an unknown history of chronic pancreatitis, or those whose pathology did not confirm pancreatic ductal adenocarcinoma (e.g., intraductal papillary mucinous neoplasm (IPMN); or pancreatic neuroendocrine tumors (PETs) were excluded. This resulted in a final cohort of 123 PDAC patients, with patient characteristics detailed in **Supplementary Table 1**. We also obtained an external validation cohort from the GEO and ArrayExpress databases (15) using the keywords “Chronic pancreatitis” and “Pancreatic cancer” or “Pancreatic Ductal Adenocarcinoma”. Ultimately, the cohorts GSE71989, E-EMBL-6 and E-MEXP-1121 were included. The GSE71989 cohort contained 8 normal samples and 13 PDAC samples. The E-MEXP-1121 cohort contained 9 normal samples and 9 CP samples, and the E-EMBL-6 cohort contained 9 CP samples and 9 PDAC samples.

### Selecting hub genes

We used the limma R package to identify differentially expressed genes (DEGs) between PDAC-AC and PDAC in the TCGA database. The data from the three cohorts, GSE71989, E-EMBL-6, and E-MEXP-1121, were analyzed for differential expression using limma, serving as external validation for the identification of key genes. The criteria for DEGs were |log2 fold change FC|≥1 and P-value <0.05. 11 hub genes were identified through CytoHubba with the hub gene SERPINE1 selected through the intersection of prognosis-related genes and hub genes in the external cohorts.

### Enrichment analysis

Functional enrichment analysis of differentially expressed genes (DEGs) was performed using the DAVID database (https://david.ncifcrf.gov/) (16). Gene Ontology (GO) and Kyoto Encyclopedia of Genes and Genomes (KEGG) analyses were performed on the DEGs with results visualized using the “clusterProfiler” and “enrichplot” R packages (17). Co-expression network analysis was performed using the online platform GeneMANIA (https://genemania.org/). Gene Set Enrichment Analysis (GSEA) was conducted to explore biological pathways using GSEA software. ClueGO was used to visualize and analyze the gene functions in a clustered network (18).

### Single-cell RNA-seq data processing

The scRNA-seq dataset used in this study was sourced from GSE212966, which included samples from 6 PDAC patients. Data analysis was conducted using the Seurat package. To ensure high-quality cells, we applied the following filtration criteria: nFeature_RNA > 1000, nFeature_RNA < 5000 and percent.mt < 10%. Highly vaPAIriable genes were identified and normalized using the SCTransform method, and batch effects were corrected using the Harmony approach (19). Dimensionality reduction was performed using Uniform Manifold Approximation and Projection (UMAP), and t-distributed Stochastic Neighbor Embedding (t-SNE), both integrated within Seurat. Differential gene expression analysis among clusters or cell types was conducted using the FindAllMarkers function, with parameters set to a p-value < 0.05, an absolute log2 fold change > 0.25, and an expression ratio > 0.1. Normalized count data from scRNA-seq and meta files containing cell type annotations were analyzed using CellChat to evaluate cell–cell interactions between Seurat clusters (20). Trajectory analyses to predict differentiation paths among various endothelial cell subtypes were conducted using Monocle3 version 1.0.0, without prior knowledge of differentiation time or direction (21).

### Tumor microenvironment and immune infiltration

The tumor immune microenvironment was assessed using single-sample Gene Set Enrichment Analysis (ssGSEA) via the “GSVA” package (version 1.52.3) in R (22). We selected the “ssgsea” method within the GSVA function for quantifying enrichment scores of individual samples. The gene sets used for this analysis were derived from the immune-related signatures reported in the study, which include 16 immune cell types and 13 immune function scores (23). TIMER2.0 (24) (http://timer.cistrome.org/) was used to estimate SERPINE1-related immune infiltration in PDAC. We applied the ESTIMATE algorithm to evaluate patients’ immune scores, stromal scores, ESTIMATE scores and tumor purity using the “estimate” R package (25). Patients were divided into “high” and “low” groups based on the median value of the corresponding variable.

### Analysis of TMB

TMB representing the number of mutations per million bases in tumor tissue, including genetic coding errors, base substitutions, insertions, and deletions (26), was calculated based on somatic mutation data from PDAC patients in the TCGA database. We also evaluated the correlation between TMB scores and gene expression.

### Clinical indicators and methylation analysis of SERPINE1

MethSurv (http://biit.cs.ut.ee/methsurv/) is an interactive portal for univariate and multivariate survival analysis using DNA methylation biomarkers from the TCGA database. Kaplan–Meier analysis was employed to assess overall survival outcomes related to SERPINE1 CpG sites across various cancers in the MethSurv database with statistical significance determined by the likelihood ratio test. The relationship between clinical indicators (such as age, tumor stage, etc.) and SERPINE1 expression levels was analyzed using the UALCAN database.

### Analysis of immune checkpoint and drug sensitivity

Immunophenoscores (IPS) for PDAC patients were obtained from The Cancer Immunome Atlas (TCIA, https://tcia.at/home) database. We also explored the response to chemotherapy drugs using the CellMiner database (27). The IMvigor210 dataset, which includes clinical information, transcriptome data and immunotherapy outcomes for 348 urothelial cancer patients, was analyzed using the IMvigor210CoreBiologies R package (17). Patients were categorized into four subgroups based on their response to immunotherapy, progressive disease (PD), stable disease (SD), partial response (PR) and complete response (CR).

### The correlation between expression of SERPINE1 and immunotherapy

The ICB resistance score was calculated using the Tumor Immune Dysfunction and Exclusion (TIDE) algorithm (28). To predict the response to immune checkpoint blockade (ICB), the Estimate Systems Immune Response (EaSIeR) method was devised, taking into account the entire tumor microenvironment (29).

### Molecular docking

AutoDock Vina 1.1.2 software was employed to calculate the binding mode of the drug to SERPINE1. The crystal structure of SERPINE1 (PDB ID: 6ZRV) was obtained from the Protein Data Bank (https://www.rcsb.org/). Solvents and original ligands were removed, and hydrogen atoms were added with corresponding atomic charges. For SERPINE1, a grid box with dimensions of 80 × 80 × 80 Å3 was established, encompassing the active binding sites. All protein and chemical structures were converted to PDBQT format using AutoDock Tools 1.5.6 prior to molecular docking.

### The construction of the nomogram

We incorporated clinical parameters to assess factors related to survival outcomes. Our analysis revealed that Age, Lymph Node Metastasis (LNM), and SERPINE1 were independent risk factors for prognosis in both univariable and multivariable Cox regression analyses. Using these independent risk factors, a nomogram was developed with the ‘rms’ R package. The nomogram’s performance was evaluated through calibration plots and ROC curves.

### Collecting clinical information and demonstrating the hub gene

We enrolled 13 patients with pancreatic ductal adenocarcinoma (PDAC) and obtained peripheral blood samples from 8 healthy controls for clinical validation. Four milliliters of peripheral blood were collected from PDSC patients before surgery. Serum SERPINE1 levels were measured using an enzyme-linked immunosorbent assay (ELISA) following the manufacturer’s instructions (Signalway Antibody, USA). We compared SERPINE1 expression levels between normal and PDAC groups. The Shapiro-Wilk test was used to assess data normality, indicating a non-normal distribution. The Mann-Whitney U test was then used to compare SERPINE1expression between the two groups. Additionally, clinical information and preoperative laboratory data, including age, gender, neutrophil count, and fibrinogen levels, were collected.

Immunohistochemistry (IHC) was conducted to validate expression in tissues, using samples from 5 normal pancreases (obtained from liver transplant donors) and 10 PDAC patients. Tissue sections underwent deparaffinization and a ten-minute boiling process in sodium citrate buffer for microwave-based antigen retrieval, followed by overnight incubation with an anti-SERPINE1 antibody (Abcam, USA, 1:200) at 4 °C. A biotinylated secondary antibody (goat–anti‐mouse–IgG) was then added for 30 minutes. This study was conducted in accordance with the Declaration of Helsinki (revised in 2013) and was approved by the Ethics Committee of Beijing Chaoyang Hospital (No. 2020-D-301).

### Cell culture and lentiviral transfection

Pancreatic cell lines PANC-1 and CFPAC-1 were obtained from Shanghai Jinyuan Biotechnology Co., Ltd. All cell types were maintained in DMEM and IMDM medium supplemented with 10% fetal bovine serum (FBS) and 1% penicillin-streptomycin, and incubated at 37 °C in a humidified atmosphere containing 5% CO_2_. Lentiviral vectors for sh-Ctrl and sh-SERPINE1 were designed and synthesized by Genechem Co., Ltd. (Shanghai, China). Pancreatic cancer cells were transduced with these vectors. Cells were plated in 6-well plates, and once they reached approximately 30% confluency, lentiviral particles (PANC-1 MOI = 20; CFPAC-1 MOI = 50) carrying either the target sequences or controls were added in the presence of 5 μg/mL polybrene (Sigma-Aldrich). After 24 hours of incubation, the medium containing viral particles was replaced with fresh culture medium. Stable cell lines expressing the target genes were subsequently selected using puromycin. The efficiency of gene silencing or overexpression was confirmed through Western blot analysis.

### Western blot

Total protein was extracted from Pancreatic cells using RIPA lysis buffer (Beyotime), supplemented with protease and phosphatase inhibitors. After centrifugation, the supernatants were collected for downstream analyses. Protein concentrations were determined with a BCA assay kit (Beyotime). For Western blotting, equal amounts of protein (20 µg per sample) were separated by SDS-PAGE and transferred onto PVDF membranes (Millipore). Membranes were blocked with 5% non-fat milk at room temperature for 1 hour and then incubated overnight at 4°C with the appropriate primary antibodies. Following three 10-minute washes with TBST, membranes were incubated with HRP-conjugated secondary antibodies for 1 hour at room temperature. After a second round of TBST washes, protein signals were detected using enhanced chemiluminescence (ECL) reagents (Biosharp) and visualized with a gel imaging system. Densitometric analysis of the bands was performed using ImageJ software.

### CCK-8 assay

Pancreatic cells were seeded into 96-well plates at a density of 5 × 10³ cells per well and allowed to adhere overnight. After applying the experimental treatments, cell proliferation was evaluated at 0, 24, 48, and 72 hours using the Cell Counting Kit-8 (CCK-8, Beyotime). At each time point, 10 μL of CCK-8 reagent was added to each well, followed by a 2-hour incubation at 37°C. Absorbance was then measured at 450 nm using a microplate reader. All conditions were performed in triplicate to ensure data reproducibility. Proliferation kinetics were analyzed by calculating relative growth rates based on absorbance values over time.

### Calcein-AM/PI double stain

Cell viability and death were assessed using a dual-fluorescence Calcein-AM/propidium iodide (PI) staining kit (Biosharp). For this analysis, 2 × 10^5^ cells were seeded into each well of 6-well plates. After completing the designated treatments, the culture medium was carefully removed and replaced with a staining solution containing 2 μM Calcein-AM and 4.5 μM PI. To preserve fluorescence integrity, the plates were shielded from light and incubated at 37°C for 30 minutes. Following incubation, cells were gently washed twice with phosphate-buffered saline (PBS) under mild agitation. Fluorescence imaging was subsequently performed using a fluorescence microscope, where live cells exhibited green fluorescence and dead cells appeared red. Quantification of cell death was carried out using ImageJ software by calculating the percentage of PI-positive cells relative to the total number of cells.

### Wound healing assay

Pancreatic cells were seeded into 6-well plates and cultured until reaching approximately 90% confluency. A linear scratch was then created across the cell monolayer using a sterile pipette tip. After removing cellular debris with PBS washes, the cells were incubated in serum-free DMEM and IMDM medium. Wound closure was monitored by capturing microscopic images at 0 hours and after 24 hours of incubation. Cell migration was assessed by measuring changes in wound width using ImageJ software, based on the acquired images.

### Statistical analysis

All statistical analyses, excluding those related to sequencing data, were performed using SPSS software (version 24.0; IBM, USA) and GraphPad Prism (version 9.0; GraphPad Software, USA). The distribution of continuous variables was assessed for normality. Data with a normal distribution are reported as mean ± standard deviation ( $\bar{\text{x}}\pm \text{SD}$). Comparisons between two groups were made using the Student’s t-test, while comparisons among multiple groups were assessed using one-way ANOVA, followed by *post hoc* t-tests for pairwise comparisons. Categorical variables are presented as percentages (%), and group differences were evaluated using the chi-square (χ²) test. For data not conforming to a normal distribution, non-parametric methods, such as the Mann-Whitney U test (rank-sum test), were employed. To evaluate the prognostic significance of SERPINE1, Kaplan-Meier survival curves were generated to compare overall survival (OS) and progression-free survival (PFS) across different expression levels, with statistical significance assessed via the log-rank test. Multivariate Cox proportional hazards regression was conducted to identify independent prognostic factors. All statistical tests were two-sided, with a p-value < 0.05 considered indicative of statistical significance.

## Results

### Screen out DEGs and functional enrichment analysis

**Figure 1:**
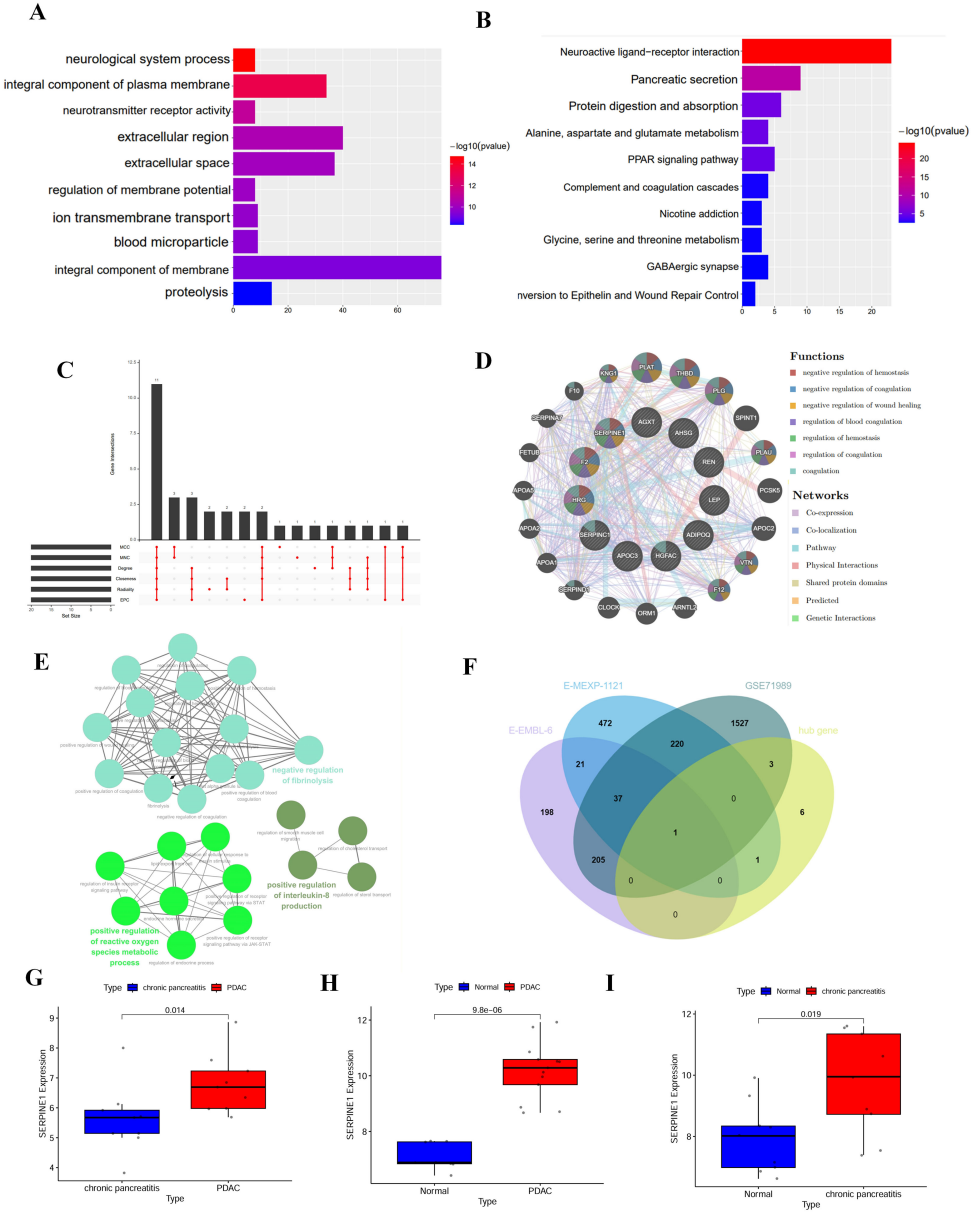
Screening and identification of hub genes. **(A, B)** The enrichment analysis results of GO and KEGG Pathway of differentially expressed genes (DEGs). **(C)** Venn diagram displaying the intersection of hub genes identified by seven different algorithms. **(D)** Interaction network of 11 hub genes analyzed using the GeneMANIA database. **(E)** Network visualization of GO terms associated with the 11 hub genes, created using the Cytoscape software. Nodes represent GO terms, and edges represent the strength of association between terms. **(F)** Venn diagram illustrating the overlap between DEGs from external cohorts and the identified hub genes dataset. **(G–I)** Expression patterns of the gene SERPINE1 in three independent cohorts: E-MEXP-1121, GSE71989, and E-EMBL-6. Expression levels were compared using “limma” package. Statistical significance was determined p-value < 0.05.

We retrieved transcriptome sequencing data and clinical information for 178 PDAC patients from the TCGA database. Among them, 110 patients had no history of chronic pancreatitis, while 13 had a history of chronic pancreatitis. Our study ultimately included 123 patients diagnosed with PDAC. **Supplementary Table 1** details the clinicopathological features of PDAC patients with and without chronic pancreatitis, showing no statistically significant differences between the two groups. In total, we identified 201 differentially expressed genes (DEGs). We conducted GO annotation and KEGG pathway enrichment analyses on these DEGs. GO analysis indicated that these 201 genes were associated with processes related to the neurological system, the integral component of the plasma membrane, neurotransmitter receptor activity, and the extracellular region (**Figure 1A**). KEGG analysis suggested correlations with pathways involving neuroactive ligand-receptor interaction, pancreatic secretion and protein digestion and absorption (**Figure 1B**). The volcano plot illustrated differential gene expression, revealing 65 upregulated and 136 downregulated genes (**Supplementary Figure 2A**).

### SERPINE1-centered network links inflammation and oncogenic pathways in PDAC

We used Cytoscape to assemble a PPI network of the DEGs, comprising 131 nodes and 214 interaction pairs (**Supplementary Figure 1A**). Through six cytoHubba plug-in algorithms, we identified the top 20 genes (**Supplementary Table 2**). Venn diagrams revealed 11 hub genes: HRG, AGXTF2, SERPINC1, AHSG, REN, APOC3, HGFAC, SERPINE1, LEP, ADIPOQ, NCF2 and TLR2 (**Figure 1C**). **Supplementary Table 3** provides their full names and functions. We explored the co-expression network and associated functions of these hub genes using GeneMANIA, which indicated 54.80% co-expression, 10.78% physical interactions, 12.81% co-localization, 8.57% shared protein domains, and 11.28% pathway association (**Figure 1D**). GO analysis using GlueGo revealed that these hub genes were enriched in various biological processes, including the positive regulation of interleukin (IL)-8 production, negative regulation of fibrinolysis, and positive regulation of the reactive oxygen species metabolic process (**Figure 1E**). We performed differential analyses on external cohorts (E-MEXP-1121, GSE71989, and E-EMBL-6), which included normal, chronic pancreatitis, and PDAC samples. The volcano plot depicted DEGs totaling 752, 1993, and 462, respectively (**Supplementary Figures 2B–D**). By intersecting these DEGs, we identified a central hub gene, SERPINE1 (**Figure 1F**). The upregulation of SERPINE1 was consistently observed across the E-MEXP-1121, GSE71989, and E-EMBL-6 databases, suggesting a potential pivotal role for SERPINE1 in inflammation and tumorigenesis (**Figures 1G–I**). TIMER2 analysis showed that SERPINE1 expression in tumor tissues exceeded that in normal tissues for BRCA, COAD, ESCA, GBM, HNSC, READ, KIRC, STAD, and THCA, whereas it was lower in UCEC, LIHC, KIRP, SKCM, and KICH tumors (**Supplementary Figure 2E**).

**Figure 2:**
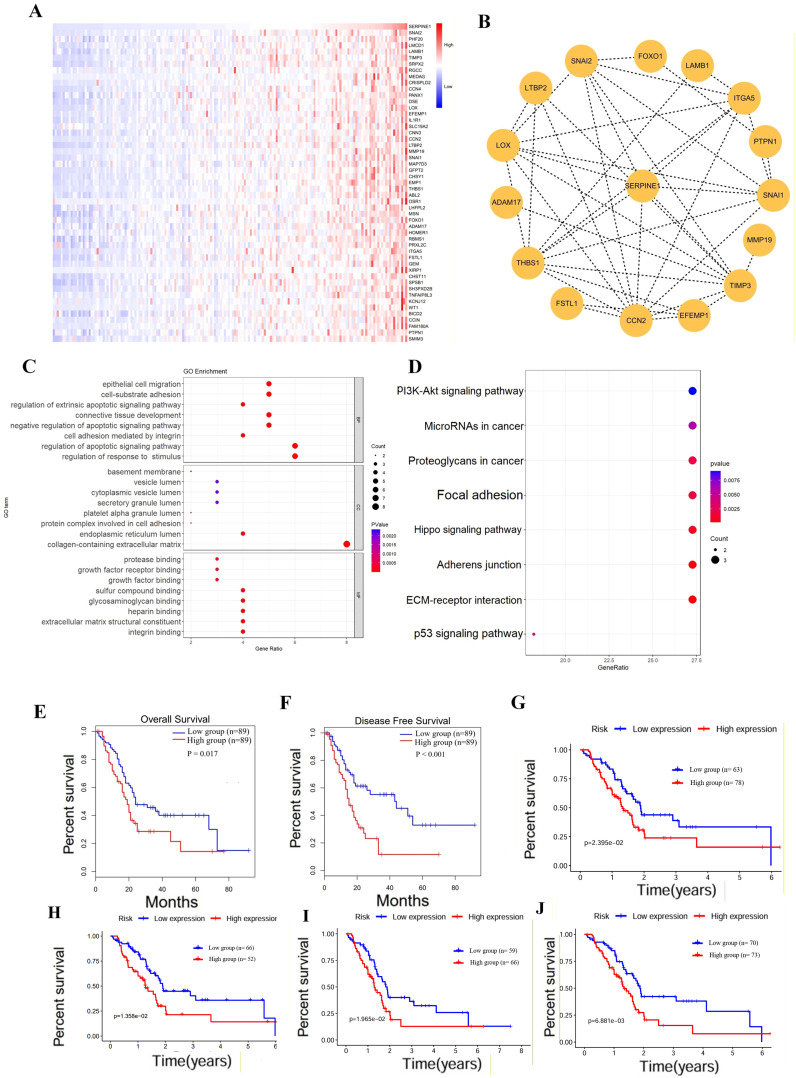
Validation the SERPINE1 expression. **(A)** Top fifty genes co-expressed with SERPINE1, identified through co-expression analysis. **(B)** Genes interacting with SERPINE1 were identified through the String database. **(C, D)** GO and KEGG enrichment analysis performed on genes co-expressed with SERPINE1, utilizing the DAVID database. **(E, F)** OS and DFS analysis in patients with high versus low SERPINE1 expression, using Kaplan-Meier curves and log-rank tests. **(G–J)** Survival analysis of SERPINE1 expression in different clinical subgroups including high histological grades (G2/3), older age (≥60 years), lymph node metastasis (N1), and advanced TNM staging (Stage 3/4), utilizing Kaplan-Meier curves and log-rank tests.

To investigate the molecular mechanism of SERPINE1 in PDAC, we obtained 100 SERPINE1-related genes from the TCGA database through correlation analysis, displaying the top 50 (**Figure 2A**). Additionally, we identified 15 SERPINE1-binding proteins from the STRING database (**Figure 2B**). GO and KEGG enrichment analyses on SERPINE1 and its binding proteins suggested that SERPINE1 is involved in regulating cellular response to growth factor stimulus, apoptotic signaling pathway, cell adhesion mediated by integrin, and other biological processes (BP). The cellular components (CC) related to SERPINE1 included the collagen-containing extracellular matrix, protein complexes involved in cell adhesion and the endoplasmic reticulum lumen. Molecular function (MF) analysis indicated SERPINE’s involvement in integrin binding, extracellular matrix structural constituent, heparin binding, and other processes (**Figure 2C**). KEGG enrichment analysis showed that SERPINE1 is associated with numerous signaling pathways, including ECM-receptor interaction, adherens junction, Hippo signaling, proteoglycans in cancer, p53 signaling, microRNAs in cancer, and PI3K-Akt signaling pathways (**Figure 2D**).

### Prognostic value of SERPINE1 in PDAC

We conducted a comparative analysis of several clinical features, including histological grading, age, lymph node metastasis, and TNM staging, to elucidate the differences between two groups characterized by high and low SERPINE1 expression levels. Our investigation, revealed that patients with elevated SERPINE1 expression exhibited higher histological grades (G2/3), younger age (41–60 years), increased likelihood of lymph node metastasis (N1), and advanced TNM staging (Stage 2) (**Supplementary Figures 3A–D**). We performed survival analysis to assess the prognostic significance of SERPINE1 in PDAC. The Kaplan-Meier (K-M) plot indicated a correlation between elevated SERPINE1 expression and unfavorable prognosis, as well as shorter DFS (**Figures 2E, F**). Additionally, our investigation revealed that high SERPINE1 expression was associated with a poorer prognosis compared to the low-expression group across various clinical subgroups. These subgroups included individuals with higher histological grades (G2/3), older age (≥60 years), higher likelihood of lymph node metastasis (N1), and more advanced TNM staging (Stage 3/4) (**Figures 2G–J**).

### Genetic alterations and promoter methylation analysis of SERPINE1

**Figure 3:**
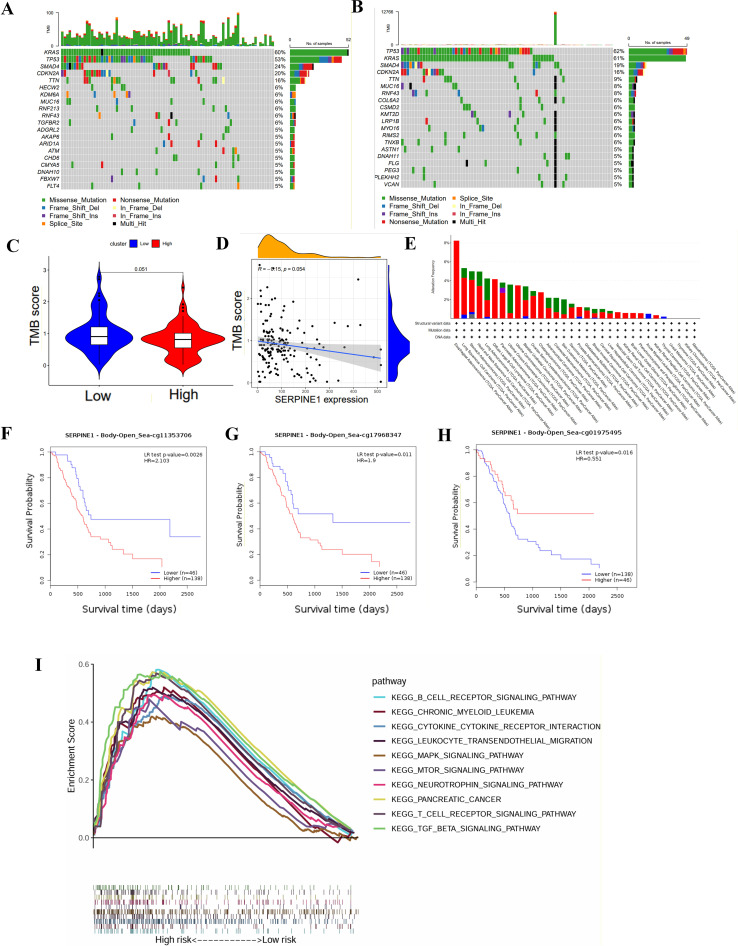
Genetic alterations and promoter methylation analysis of SERPINE1. **(A, B)** Waterfall plots showing the most frequently mutated genes in the low SERPINE1 expression group. **(C, D)** Correlation between tumor mutational burden (TMB) scores and SERPINE1 expression levels, analyzed using Spearman correlation. **(E)** Pan-cancer analysis of SERPINE1 alteration frequency across different cancer types. **(F–H)** Promoter methylation levels of SERPINE1 in relation to tumor stage, tumor grade, and TP53 mutation status, analyzed using Kruskal-Wallis and Mann-Whitney U tests. **(I)** Gene Set Enrichment Analysis (GSEA) highlighting the top 10 enriched pathways in the high SERPINE1 expression group.

Analyses of mutation profiles based on SERPINE1 expression suggested that the high SERPINE1 expression group showed slightly higher frequencies of TP53 (62%) and KRAS (61%) mutations compared with the low-expression group (53% and 60%, respectively), these differences did not reach statistical significance (**Figures 3A, B**). The high SERPINE1 expression group also exhibited a tendency towards lower TMB scores, albeit with a p-value of 0.051 (**Figure 3C**). Correlation analysis revealed a negative association between TMB and SERPINE1 expression (**Figure 3D**). We explored the genetic alteration of SERPINE1 in various tumors using cBioPortal. The alteration frequency of SERPINE1 was 3.8%, including amplifications, structural variants and mutations (**Figure 3E**). DNA methylation crucial for cell development, and differentiation, and disease pathogenesis, showed aberrant patterns in cancers. We investigated the prognostic implications of DNA methylation at CpG sites within the SERPINE1 gene using data from the Methsurv database. The findings suggested that DNA methylation levels at 15 CpG sites in SERPINE1 were linked to cancer prognosis, including cg11353706, cg17968347, and cg01975495. (p < 0.05, **Supplementary Table 4**, **Figures 3F–H**). Promoter methylation analysis conducted via UALCAN, revealed that higher promoter methylation was associated with lower tumor stage (Stage 1) and tumor grade (Grade 1) (**Supplementary Figures 3E, F**). Additionally, the promoter methylation level of SERPINE1 was significantly higher in PDAC patients with TP53 mutations compared to those without (**Supplementary Figure 3J**). To enhance our understanding of SERPINE1’s biological functions, we conducted GSEA. Our findings revealed a significant correlation between elevated SERPINE1 expression and pathways associated with tumor progression and inflammation, including the pathways in mtor signaling, pancreatic cancer, Notch signaling, MAPK signaling, T cell receptor signaling, B cell receptor signaling, leukocyte transendothelial migration, and neurotrophin signaling, suggesting that SERPINE1 may be implicated in both tumorigenesis and inflammation processes (**Figure 3I**).

### Immune infiltration and tumor microenvironment

**Figure 4:**
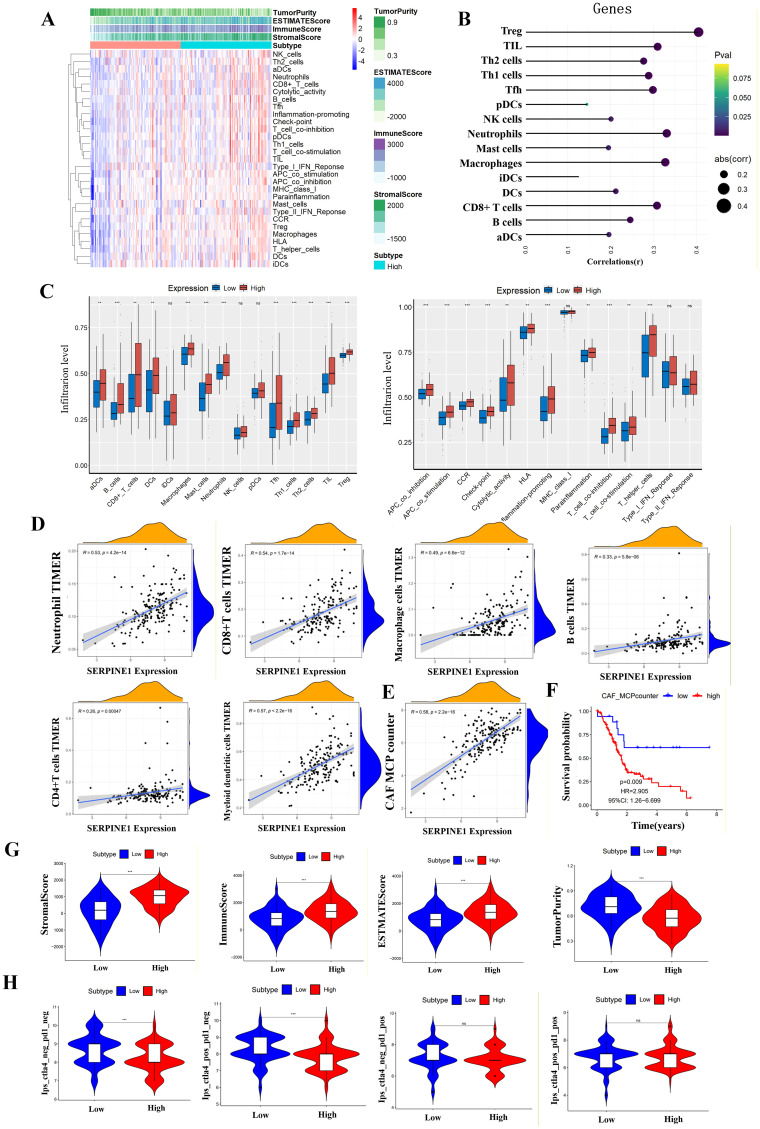
The analysis of tumor immune microenvironment in PDAC. **(A)** Heatmap illustrating the differential abundance of various immune cell types between high and low SERPINE1 expression groups. **(B)** Correlation analysis between SERPINE1 expression levels and specific immune cell subtypes, using Spearman correlation. **(C)** Relationship between SERPINE1 expression and immune cell subsets/functions. **(D)** Positive correlation between SERPINE1 expression and the infiltration levels of neutrophils, CD8+ T cells, macrophages, B cells, CD4+ T cells, and myeloid cells, using Spearman correlation. **(E–F)** Correlation and survival analysis of cancer-associated fibroblasts (CAFs) in relation to SERPINE1 expression, utilizing Spearman correlation and Kaplan-Meier curves with log-rank tests. **(G)** Comparison of stromal score, immune score, ESTIMATE score, and tumor purity between different SERPINE1 expression levels, using the ESTIMATE algorithm and Wilcoxon rank-sum test. **(H)** IPS score comparison between high and low SERPINE1 expression groups, using the Wilcoxon rank-sum test. ***p < 0.001; **0.001 < p < 0.01; *0.01 < p < 0.05; ns p > 0.05.

We investigated the differences in tumor immune microenvironment between high and low SERPINE1 expression groups using the ssGSEA algorithm (**Figure 4A**). SERPINE1 exhibited a positive correlation with numerous immune cells (**Figure 4B**). Elevated levels of immune cell infiltration including Neutrophils, Mast cells, and Macrophages, were observed in the high SERPINE1 expression group. Furthermore, the proportion of cells promoting inflammation was significantly greater in the high expression group. Our findings also indicated that the high SERPINE1 expression group exhibited a greater proportion of cytolytic activity and higher HLA levels compared to the low-expression group (**Figure 4C**). The infiltration of Neutrophils, T cell CD8+ cell, macrophages, B cell, T cell CD4+ cell and Myeloid cell was significantly positively correlated with SERPINE1 expression in TIMER2, consistent with our analysis (**Figure 4D**). A positive correlation was observed between cancer-associated fibroblasts (CAFs) and SERPINE1 expression (**Figure 4E**). Increased CAF infiltration was associated with poorer patient prognosis (**Figure 4F**). The Immune score, Stromal score, and ESTIMATE score were significantly higher in the high SERPINE1 expression group; while tumor purity exhibited an opposing trend (**Figure 4G**). To test the ability of SERPINE1 expression to predict ICI response, IPS analysis was conducted to determine the immunotherapeutic sensitivity of PDAC patients. The IPS_CTLA4_neg_PD1_neg and IPS_CTLA4_pos_PD1_neg scores were significantly higher in the low SERPINE1 expression group compared to the high-expression group, while no significant differences were observed in the other IPS score sets (**Figure 4H**).

### Treatment strategy and drug sensitivity in PDAC

**Figure 5:**
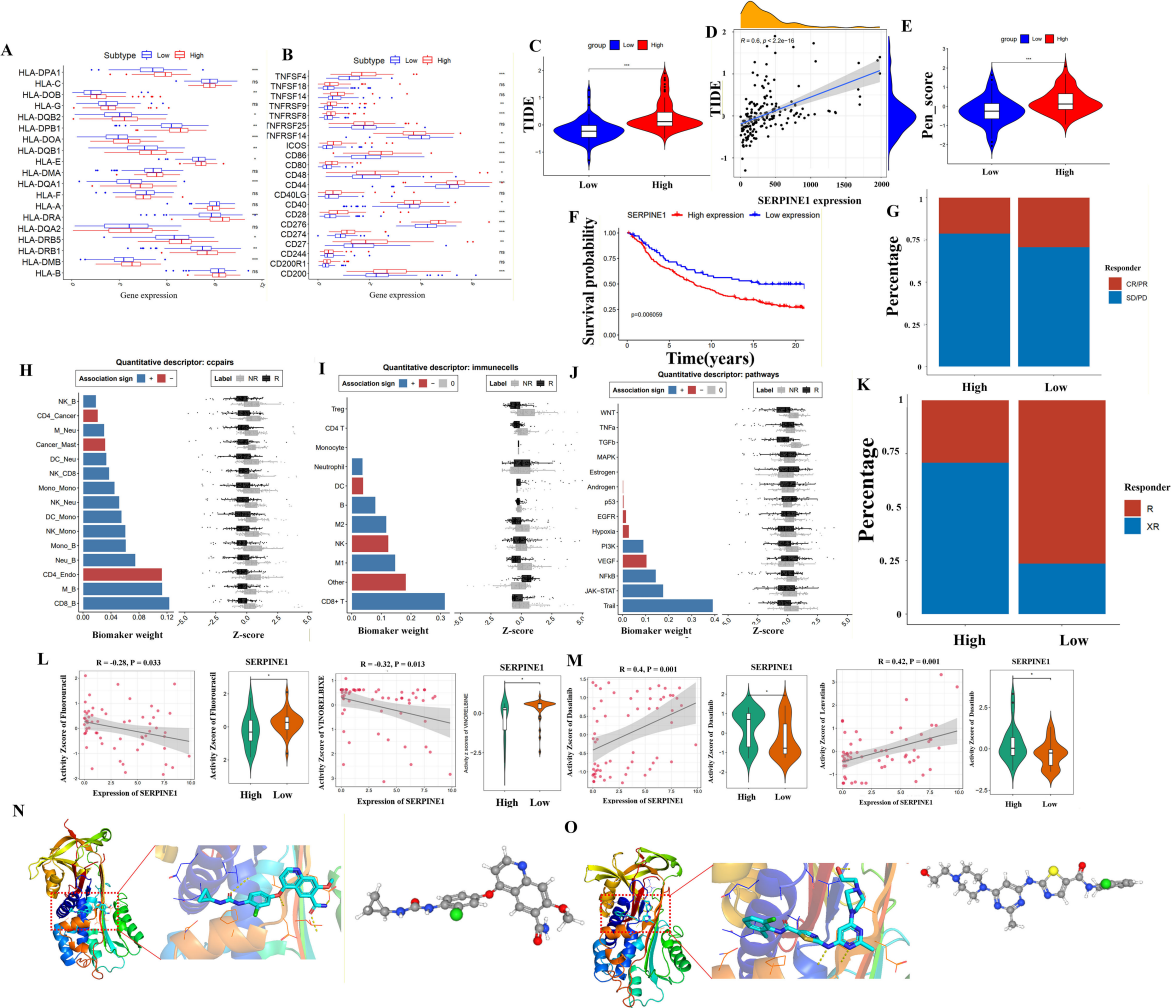
Immune analysis and drugs sensitivity. The analysis of HLA **(A)** and TNF **(B)** with SERPINE1 expression in PDAC. **(C–E)** TIDE score and Pen score comparison between high and low SERPINE1 expression groups. Statistical significance was determined using the Wilcoxon rank-sum test. **(F)** Kaplan-Meier survival curves comparing high and low SERPINE1 expression in the IMvigor210 cohort. The log-rank test was used for statistical analysis. **(G)** Bar graph showing Stable Disease/Progressive Disease (SD/PD) and Complete Response/Partial Response (CR/PR) rates in high and low SERPINE1 expression groups in IMvigor210. **(H–J)** Analysis of cell-cell interaction pairs, immune cells, and pathways in the context of immunotherapy, employing network analysis tools. **(K)** Bar graph illustrating response and non-response rates to therapy in high and low SERPINE1 expression groups in TIDE. Chi-square test was used for comparison. **(L, M)** Drug sensitivity assays comparing high and low SERPINE1 expression groups for Fluorouracil, Vinorelbine, Dasatinib, and Lenvatinib. IC50 values were calculated and compared using the Student’s t-test. **(N, O)** Molecular docking simulations and 3D structural models of Lenvatinib and Dasatinib interactions with their targets ***p < 0.001; **0.001 < p < 0.01; *0.01 < p < 0.05; ns p > 0.05.

In our study, we observed significant difference in HLA expression, particularly HLA-DPA1, HLA-DOA, and HLA-DMB, between the two groups (**Figure 5A**). Additionally, the expression of TNF and certain immune molecules also showed statistically significant differences between the high and low expression groups (**Figure 5B**). To assess the response to immunotherapy in two patient groups, we compared the TIDE scores. We found that patients with high SERPINE1 expression had higher TIDE scores compared to those with low SERPINE1 expression (**Figure 5C**). Furthermore, a positive correlation was identified between TIDE scores and SERPINE1 expression levels, suggesting that patients in the low-expression group may have a better response to immunotherapy (**Figure 5D**). This observation is further supported by the pen score (**Figure 5E**). Next, we used the IMvigor210 cohort to investigate the efficacy of immunotherapy in patients treated with anti-PD-L1 agents. As shown in **Figure 5F**, individuals with low SERPINE1 expression exhibited better survival outcomes compared to those with high SERPINE1 expression. Patients with high SERPINE1 expression had a higher proportion of SD or PD, CR or PR was more common in the low SERPINE1 expression group (**Figure 5G**). We subsequently evaluated the efficacy of EaSIeR along with cancer-type-specific machine learning models, constructed using TCGA data. These models were employed to predict immune response scores, and the probability of patient responsiveness to ICB therapy. We observed interactions between CD8+ T cells and B cells, with CD8+ T cells, and the TRAIL pathway, playing significant roles in the pharmacological treatment of PDAC (**Figures 5H–J**). Using the TIDE database to predict immune therapy response outcomes, we observed a higher proportion of responders in the low-expression group (**Figure 5K**). The efficacy of four chemotherapeutic agents was assessed, revealing that the low SERPINE1 expression group demonstrated heightened sensitivity to several conventional chemotherapeutic agents, including Fluorouracil and Vinorelbine, compared to the high SERPINE1 expression group (**Supplementary Table** Drug_predict_res and DTP_NCI60_ZSCORE). Elevated SERPINE1 expression may indicate a positive correlation with specific targeted drugs, such as Dasatinib and Lenvatinib (**Figures 5L, M**). Docking studies showed that Lenvatinib and Dasatinib bind to the SERPINE1 molecule at potential binding energies of around -6.62 and -6.96, respectively. The 3D structures of Lenvatinib and Dasatinib were explored using PubChem (**Figures 5N, O**). It should be noted that Lenvatinib and Dasatinib have not been experimentally validated to bind or inhibit SERPINE1, and therefore any docking results with these compounds are purely predictive.

### scRNA-seq identifies SERPINE1-enriched myCAF and iCAF subpopulations

**Figure 6:**
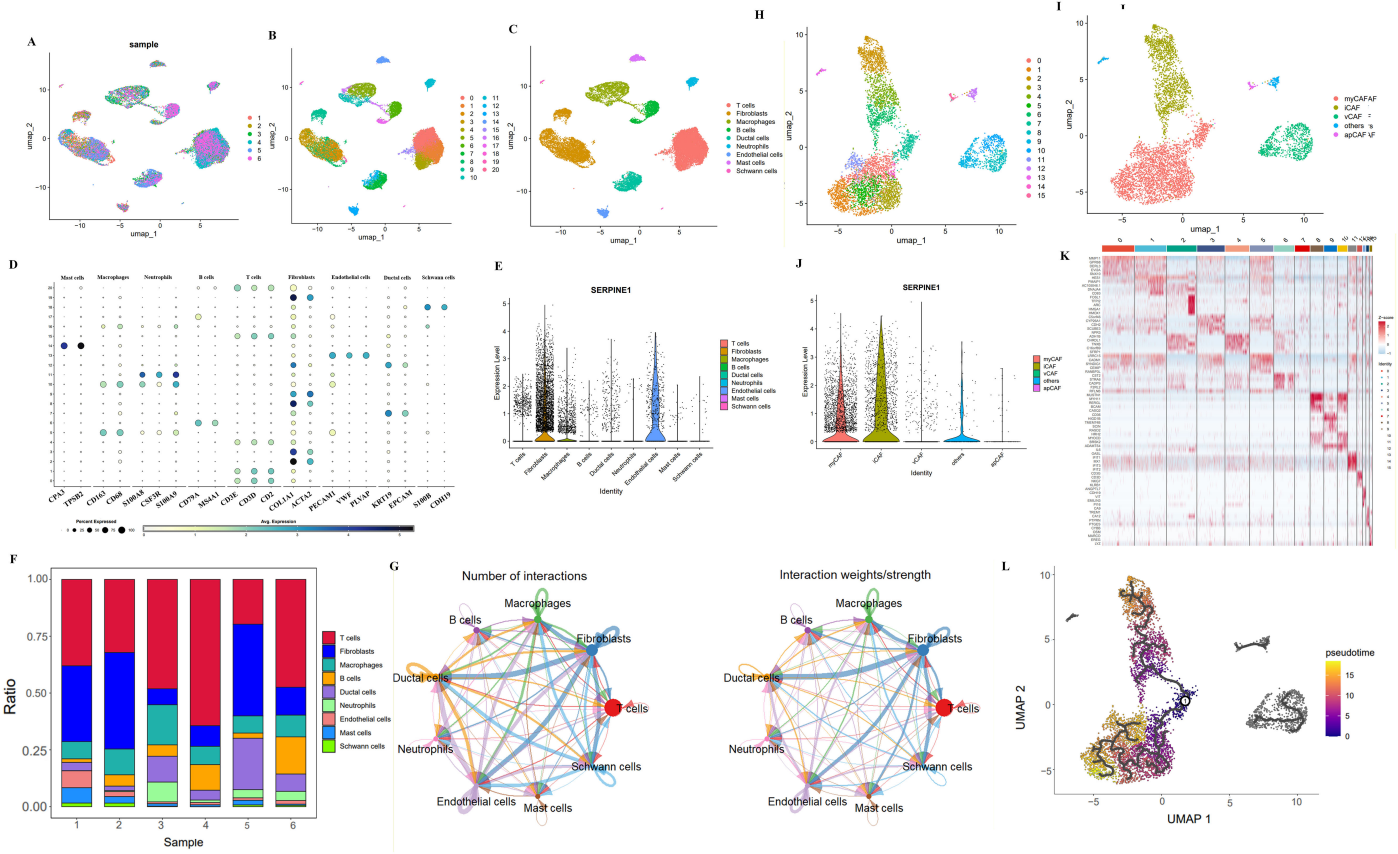
Single cell sequencing analysis. **(A)** Visualization of six samples after batch effect removal using the Harmony algorithm. **(B)** Cells clustered into 21 distinct clusters using the Louvain algorithm. **(C, D)** A total of 9 cell types was classified, such as mast cell, ductal cells and neutrophils. **(E)** Expression of SERPINE1 in fibroblasts and endothelial cells identified. **(F)** Heterogeneity among different samples, visualized using UMAP plots. **(G)** Cellular communication and interaction networks analyzed using CellChat. **(H)** Sub-clustering of fibroblasts into specific subgroups. **(I)** Identification of 5 cellular subgroups. **(J)** SERPINE1 expression predominantly in myCAF and iCAF subtypes. **(K)** Differential expression analysis identifying cluster-specific genes. **(L)** Pseudotime trajectory analysis revealing the differentiation pathways of various cell types.

In the single-cell data from six pancreatic cancer patients after batch correction (**Figure 6A**), we performed unsupervised hierarchical clustering of the single-cell gene expression profiles, identifying 21 clusters at a resolution of 1 (**Figure 6B**, **Supplementary Figure 4A**). Through literature review, molecular markers for various cells were identified, categorizing them into nine cell types (**Figures 6C, D**). SERPINE1 was predominantly expressed in fibroblasts and endothelial cells (**Figure 6E**, **Supplementary Figures 4B, C**). Analysis the cellular composition of each patient, revealed variations in the proportion of different cell types, highlighting the heterogeneity of pancreatic cancer (**Figure 6F**). CellChat was used to delineate intricate cell-to-cell communications and predict biologically significant findings from scRNA-seq data. **Figure 6G** shows the aggregated cell–cell communication network, indicating significant interactions between endothelial cells, ductal cells and fibroblasts. Given the crucial role of fibroblasts in cellular interactions, we subgrouped fibroblasts. After batch correction (**Supplementary Figure 3D**), unsupervised hierarchical clustering revealed 16 clusters at a resolution of 1.2 (**Figure 6H**, **Supplementary Figure 4E**). Using cell surface molecular markers and gene differential analysis heatmaps, fibroblasts were classified into five groups: myCAFs, iCAFs, vCAFs, apCAFs, and others (**Figures 6I, K**, **Supplementary Figure 4F**). SERPINE1 was primarily expressed in the myCAF and iCAF subgroups (**Figure 6J**). Retrograde analysis, suggested that myCAF and iCAF share a common differentiation ancestor, distinct from other CAF sources (**Figure 6L**), aligning with previous research findings (30).

### Clinical and immune correlates of SERPINE1 expression in PDAC

**Figure 7:**
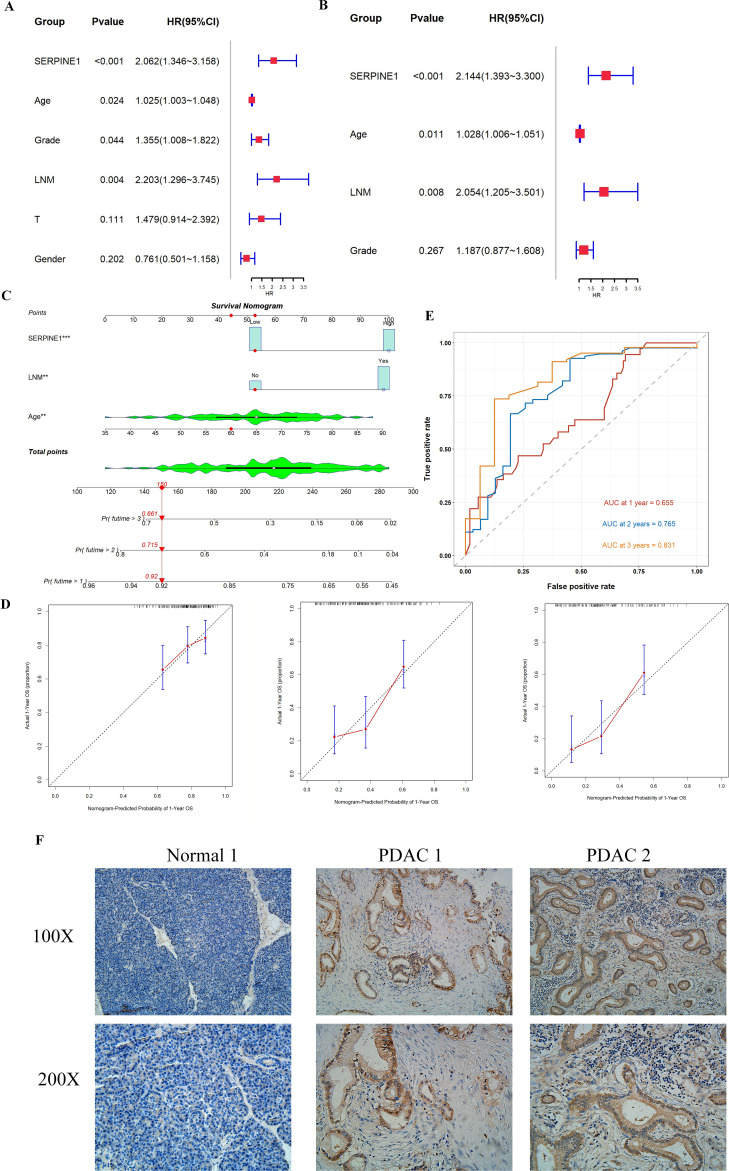
Prognostic model and experimental validation. **(A)** Forest plot of univariable Cox regression analysis for overall survival in PDAC patients. **(B)** Forest plot of multivariable Cox regression analysis incorporating age, lymph node metastasis (LNM), and SERPINE1 expression. **(C)** Nomogram predicting 1-, 2-, and 3-year survival probabilities, integrating age, LNM, and SERPINE1 expression. **(D)** Calibration curves evaluating the accuracy of the nomogram at 1, 2, and 3 years. **(E)** Receiver Operating Characteristic (ROC) curves assessing the predictive performance of the nomogram. **(F)** Immunohistochemical validation of SERPINE1 expression in PDAC tissues.

We identified age, lymph node metastasis (LNM), and SERPINE1 expression as independent prognostic risk factors through univariable and multivariate Cox regression analyses (**Figures 7A, B**). We constructed a nomogram using these three independent prognostic risk factors (**Figure 7C**). The calibration curves for 1-, 2-, and 3-year intervals showed a strong correlations between predicted and actual outcomes (**Figure 7D**). The ROC curve further indicated the commendable performance of the nomogram, with its 3-year predictive ability being more accurate than the 1- and 2-year predictions (**Figure 7E**). For clinical validation, we enrolled 13 PDAC patients and 8 healthy controls. The clinical and pathological characteristics of both groups are presented in **Supplementary Figure 5A**. No statistically significant differences were found between the groups. Serum SERPINE1 levels, assessed using ELISA, revealed significantly higher levels in PDAC patients compared to healthy controls (**Supplementary Figure 5B**). Given the positive correlation with several immune cells, including Neutrophils and Macrophages, we examined the correlation with Neutrophil counts in peripheral blood (**Supplementary Figure 5C**). The findings indicated a positive correlation between Neutrophil counts and SERPINE1 expression, suggests that elevated SERPINE1 expression may enhance Neutrophil infiltration, corroborating our previous analyses. Immunohistochemistry further confirmed high SERPINE1 expression in PDAC tissues (**Figure 7F**). As indicated in the figure, DAB-positive brown staining represents SERPINE1 expression, whereas the absence of brown coloration indicates that no discernible SERPINE1 signal was detected in that field. Our study highlights a significant association between SERPINE1 and the tumor immune microenvironment, underscoring its pivotal role in tumorigenesis.

### SERPINE1 promotes the malignant PC process *in vitro*

**Figure 8:**
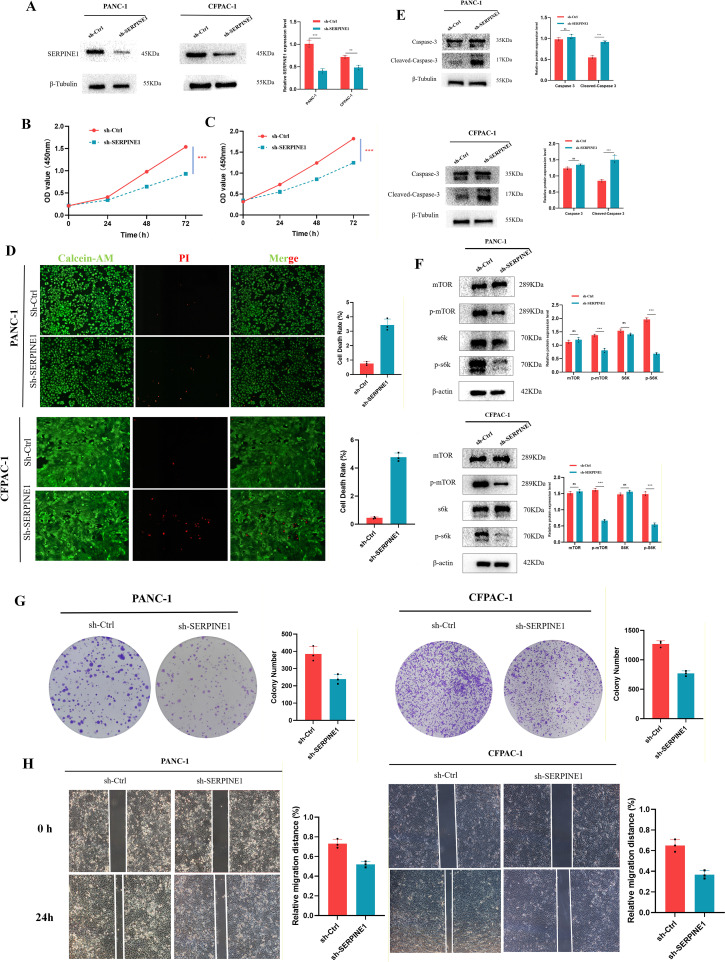
SERPINE1 promotes the PDAC process *in vitro*. **(A)** Western blot analysis of SERPINE1 protein levels in PANC-1 and CFPAC-1 cells with SERPINE1 knockdown. **(B, C)** Viability of PANC-1 and CFPAC-1 cells transfected with sh-SERPINE1. **(D)** Calcein-AM/PI staining of cell death rate in PANC-1 and CFPAC-1 cells with SERPINE1 knockdown. **(E)** Western blot analysis of cleaved caspase-3 in PANC-1 and CFPAC-1 cells. **(F)** Protein levels of mTOR, p-mTOR, S6K, and p-S6K in PANC-1 and CFPAC-1 cells after SERPINE1 knockdown. **(G)** Colony-formation assay showing proliferation in PANC-1 and CFPAC-1 cells with SERPINE1 knockdown. **(H)** Wound healing assay showing migration in PANC-1 and CFPAC-1 cells with SERPINE1 knockdown ***p < 0.001; **0.001 < p < 0.01; *0.01 < p < 0.05; ns p > 0.05.

To elucidate the role of SERPINE1 in pancreatic cancer progression and metastasis, we modulated its expression in PANC-1 and CFPAC-1 cell lines using lentiviral transduction. SERPINE1 levels were knocked down through siRNA infection (**Figure 8A**). CCK-8 assays revealed that SERPINE1 knockdown significantly inhibited cell proliferation (**Figures 8B, C**). Importantly, SERPINE1 silencing in PANC-1 and CFPAC-1 cell lines increased apoptotic rates after 24 hours (**Figure 8D**). These findings were corroborated by immunoblotting, which showed increased cleaved caspase-3 in SERPINE1-deficient PANC-1 and CFPAC-1 cells (**Figure 8E**). GSEA revealed a strong association between SERPINE1 expression and activation of the mTOR signaling pathway. Specifically, elevated SERPINE1 expression correlated with enhanced mTOR pathway activity. To further validate this finding, we examined key components of the mTOR pathway. Notably, SERPINE1 knockout led to a marked reduction in phosphorylated mTOR (p-mTOR) and phosphorylated S6K (p-S6K) levels. These results suggest that SERPINE1 may modulate tumor biological behavior, at least in part, through regulation of mTOR pathway activation (**Figure 8F**). Functional analyses using Colony-formation assay and wound healing assays further demonstrated that SERPINE1 knockdown impaired the migratory capacity of PANC-1 and CFPAC-1 cell (**Figures 8G, H**). Collectively, these data support a pro-tumorigenic role for SERPINE1, accelerating the progression of PDAC *in vitro*.

## Discussion

Chronic pancreatitis (CP) is a well-established risk factor for pancreatic ductal adenocarcinoma (PDAC). One study reported that the cumulative incidence of PDAC within two years among patients with CP was 16.16%, with the risk of developing pancreatic cancer increasing nearly eightfold during the first five years after CP diagnosis (31). In our study, we focused on the SERPINE1 gene, selected based on its association with both prognosis and hub genes, suggesting a potentially important role in tumor progression and inflammation. We comprehensively examined the relationship between SERPINE1 expression and the tumor microenvironment (TME) in PDAC. Our results showed that SERPINE1 was significantly upregulated in PDAC tissues compared to adjacent normal tissues, and higher expression correlated with poorer overall survival. Functionally, SERPINE1 promoted tumor cell proliferation and migration. Notably, its expression was closely linked to an immunosuppressive TME, marked by increased infiltration of cancer-associated fibroblasts and tumor-associated macrophages. These findings indicate that SERPINE1 may act as a key mediator connecting stromal remodeling and immune suppression in pancreatic cancer.

SERPINE1 encodes endothelial plasminogen activator inhibitor 1 (PAI-1), a member of the serine protease inhibitor (serpin) family that inhibits tPA and uPA, thereby regulating fibrinolysis (32).

Previous studies have shown that SERPINE1 not only directly promotes tumor cell proliferation and migration but also, as a multifunctional molecule, contributes to extracellular matrix (ECM) degradation, angiogenesis, and fibrosis, thereby facilitating tumor progression and metastasis (33). In highly desmoplastic malignancies, cancer-associated fibroblasts (CAFs) are a major mesenchymal cell population in the tumor microenvironment (TME), and their abundance is associated with poor prognosis. CAFs drive stromal remodeling into a dense, fibrotic matrix while secreting factors that sustain cancer stem-like properties, enhance tumor cell survival, promote invasive growth and metastasis, and reduce sensitivity to chemotherapy. Consequently, tumors with pronounced stromal fibrosis are more prone to drug resistance and recurrence (34). CAFs secrete high levels of SERPINE1, which inhibits plasminogen activation and stabilizes ECM components, generating a stiff, hypovascular stroma. This remodeled stroma can physically hinder immune cell infiltration and functionally suppress cytotoxic immunity through hypoxia and cytokine signaling (14). Shuhai Chen and colleagues reported that CAFs promote M2 polarization of tumor-associated macrophages (TAMs) via CXCL12, leading these TAMs to secrete PAI-1. The TAM-derived PAI-1 then enhances hepatocellular carcinoma (HCC) malignancy through epithelial–mesenchymal transition (EMT), highlighting the CAF/TAM/PAI-1/CXCL12 axis as a potential therapeutic target (35). Similarly, in the esophageal squamous cell carcinoma (ESCC) TME, CAF-like cells induced by co-culture of mesenchymal stem cells (MSCs) with cancer cells express high levels of PAI-1. Through LRP1-mediated activation of the AKT/ERK1/2 pathway, PAI-1 promotes the migration and invasion of both cancer cells and macrophages, correlating with poor prognosis and identifying the PAI-1/LRP1 axis as a promising therapeutic target (36). In this study, we found that SERPINE1 was highly expressed in tumor tissues, and its elevated expression was associated with increased infiltration of cancer-associated fibroblasts (CAFs). Notably, patients with high CAF infiltration exhibited poorer prognosis. Consistently, single-cell RNA sequencing analysis revealed similar findings, showing that SERPINE1 was predominantly expressed in CAFs. These results suggest that, in PDAC—a tumor characterized by pronounced stromal fibrosis—both SERPINE1 and CAFs play a critical role in shaping the tumor microenvironment.

SERPINE1 also plays a key role in regulating immune cell infiltration within tumors. Previous studies suggest that the limited efficacy of immune checkpoint inhibitors (ICIs) in pancreatic ductal adenocarcinoma (PDAC) largely stems from a fibrotic and inflammatory tumor microenvironment (TME). This environment is shaped by cancer-associated fibroblast (CAF)-mediated remodeling of the extracellular matrix (ECM) and by the infiltration and polarization of tumor-associated macrophages (TAMs). These processes contribute to T-cell exclusion and are central mechanisms through which the stroma mediates resistance to immunotherapy (37–39). SERPINE1 interacts with lipoprotein receptor-related protein 1 (LRP1), promoting M2 macrophage polarization. This, in turn, reduces CD8^+^ T-cell infiltration and impairs their cytotoxic function within the TME, weakening antitumor immunity. In advanced gastric cancer (GC), high SERPINE1 expression correlates with an increased risk of recurrence following treatment with ICIs, such as PD-1 blockade. Importantly, combining SERPINE1 inhibition with PD-1 blockade has shown synergistic antitumor effects (40). Mechanistically, tumor-derived SERPINE1 drives TAM M2 polarization through the let-7g-5p/SOCS7/STAT3 axis, promoting GC progression and therapeutic resistance (41). Falcomatà et al. provided spatially resolved mechanistic and functional evidence that tumor cell expression of SERPINE1 (PAI-1) and SERPINB2 (PAI-2) in PDAC generates a fibrin(ogen)-rich ECM. This ECM recruits and programs immunosuppressive macrophages while excluding and exhausting CD8^+^ T cells, ultimately promoting tumor growth and resistance to PD-1 blockade (42). High PAI-1 expression by tumor and stromal cells activates the JAK/STAT pathway, upregulating PD-L1 and enhancing immunosuppressive signaling. Targeting PAI-1 reduces the accumulation of immunosuppressive cells, increases cytotoxic T lymphocyte (CTL) infiltration, and restores immune surveillance. When combined with ICIs, such as anti-PD-L1 antibodies, PAI-1 inhibition further amplifies TME immune activation and significantly enhances tumor regression (43). These findings suggest that combining SERPINE1 blockade with immunotherapy or stromal-modulating strategies represents a promising direction for future research. We systematically analyzed the differences between high and low SERPINE1 expression groups in terms of immune cell infiltration and response to immunotherapy. We observed significant differences in immune cell populations, particularly macrophages and neutrophils, as well as in immunotherapeutic outcomes. *In vitro* experiments further demonstrated that SERPINE1 knockout can promote apoptosis and inhibit the mTOR pathway, suggesting that SERPINE1 may influence tumor cell behavior through these mechanisms. However, the tumor microenvironment is highly complex, and therapy resistance involves interactions among stromal and immune cells. Therefore, further in-depth studies are warranted to fully elucidate the role of SERPINE1 in tumor progression and treatment response.

While our study highlights the role of SERPINE1 in shaping the tumor immune microenvironment, several limitations should be acknowledged. First, transcriptomic and clinical data derived from public databases are not without flaws; patient follow-up may be incomplete, and tissue sample collection may lack standardization. Second, *in vivo* validation using SERPINE1-targeting drugs is needed to determine whether inhibiting SERPINE1 can reverse immunosuppression and enhance immunotherapy efficacy. Third, although SERPINE1 is highly expressed in tumor-associated fibroblasts, the contribution of CAFs to TME remodeling in PDAC requires further experimental confirmation. Therefore, current evidence is insufficient to establish SERPINE1 as a clinical biomarker, and extensive research is needed to explore potential therapeutic strategies for PDAC patients.

## Conclusions

In summary, our integrative analyses pinpoint SERPINE1 as a central molecular driver at the intersection of inflammation, tumor progression, and immune modulation in PDAC. High SERPINE1 expression was not only closely associated with extensive immune cell infiltration but also predicted poor responsiveness to conventional chemotherapeutics, underscoring its role in shaping an immunosuppressive and therapy-resistant microenvironment. Functional interrogation through CRISPR-mediated knockout in PDAC cell lines demonstrated that SERPINE1 is indispensable for sustaining malignant phenotypes, including proliferation, migration, and survival, in part through regulation of the mTOR signaling axis. Together, these findings elevate SERPINE1 from a prognostic biomarker to a translationally actionable target, opening new avenues for therapeutic intervention and rational drug development in pancreatic cancer.

## Data Availability

The original contributions presented in the study are included in the article/**Supplementary Material**. Further inquiries can be directed to the corresponding authors.

## Glossary

CP: chronic pancreatitis
PDAC: pancreatic ductal adenocarcinoma
PDAC-PC: PDAC with history of chronic pancreatitis
TCGA: The Cancer Genome Atlas
TME: Tumor Microenvironment
PC: pancreatic cancer
MDSCs: myeloid-derived suppressor cells
OS: overall survival
tPA: tissue plasminogen activator
IPMN: intraductal papillary mucinous neoplasm of the pancreas
PETs: pancreatic neuroendocrine tumors
DEGs: differential expressed genes
ICIs: immune checkpoint inhibitors
GO: Gene Ontology
KEGG: Kyoto Encyclopedia of Genes and Genomes
GSEA: Gene Set Enrichment Analysis
ssGSEA: single-sample Gene Set Enrichment Analysis
PD: progressive disease
IPS: Immunophenoscore
SD: stable disease
PR: partial response
CR: complete response
SD: standard deviation
ELISA: enzyme-linked immunosorbent assay
IL: Interleukin
K-M: Kaplan–Meier
PAI-1: Endothelial Plasminogen Activator Inhibitor 1
T2DM: type 2 diabetes
NETs: neutrophil extracellular traps
PADI4: peptidyl arginine deiminase-4
myCAFs: Myofibroblastic Cancer-Associated Fibroblasts
iCAFs: Inflammatory Cancer-Associated Fibroblasts
vCAFs: Vascular Cancer-Associated Fibroblasts
apCAFs: Antigen-Presenting Cancer-Associated Fibroblasts
UMAP: Uniform Manifold Approximation and Projection,
t-SNE: t-distributed Stochastic Neighbor Embedding
TIDE: Tumor Immune Dysfunction and Exclusion
IHC: immunohistochemistry
PBS: phosphate-buffered saline
