# Supplementary material for: Integrated multi-omics and single-cell analysis identify SERPINE1 as a key mediator of the inflammatory tumor microenvironment in PDAC
Source: Front Immunol. 2026 Jan 12;16:1716878. doi: 10.3389/fimmu.2025.1716878 (PMC12833254; doi:10.3389/fimmu.2025.1716878)
Supplement: Supplementary file 1 [file DataSheet1.zip › Supplementary Material/Supplementary Table.docx]

Supplementary table 1 Clinical and pathologic characteristics of 123 patients in PDAC-CP and PDAC cohorts

| Characteristics | PDAC-CP | PDAC | P |
| --- | --- | --- | --- |
| Age (Year), Mean ± SD | 61.1±10.4 | 64.7±11.4 | 0.280 |
| Gender (male/female) | 6/7 | 65/45 | 0.372 |
| Smoking (Yes/No) | 4/9 | 28/82 | 0.680 |
| Drinking (Yes/No) | 10/3 | 71/39 | 0.373 |
| Radiation therapy (Yes/No) | 2/8 | 21/63 | 0.728 |
| Tumor size (cm), Mean ± SD | 3.9±1.3 | 3.7±1.5 | 0.715 |
| Histologic grade (G1-2/G3-4) | 10/3 | 74/36 | 0.479 |
| LNM(Yes/No) | 8/5 | 83/27 | 0.279 |

Supplementary table 2 The top 20 hub genes rank in cytoHubba.

| MCC | MNC | Degree | Closeness | Radiality | EPC |
| --- | --- | --- | --- | --- | --- |
| AHSG | SERPINC1 | AHSG | LEP | LEP | AHSG |
| SERPINC1 | AHSG | SERPINC1 | AHSG | ADIPOQ | SERPINC1 |
| F2 | F2 | F2 | ADIPOQ | AHSG | HRG |
| APOC3 | HRG | LEP | SERPINC1 | APOC3 | F2 |
| APOA2 | CPA1 | HRG | F2 | SERPINE1 | APOC3 |
| HRG | APOC3 | CPA1 | APOC3 | NTRK2 | ADIPOQ |
| SERPINA7 | ADIPOQ | ADIPOQ | PAX6 | PAX6 | LEP |
| HGFAC | LEP | APOC3 | NTRK2 | HRG | HGFAC |
| CPA1 | APOA2 | PAX6 | HRG | F2 | APOA2 |
| AGXT | SERPINA7 | NTRK2 | SERPINE1 | REN | SERPINE1 |
| CPA2 | HGFAC | REN | REN | GIP | REN |
| CELA3A | CPA2 | HGFAC | HGFAC | SERPINC1 | SERPINA7 |
| PRSS3P2 | SERPINE1 | SERPINE1 | GIP | HGFAC | AGXT |
| ADIPOQ | AGXT | AGXT | CALB1 | CALB1 | PAX6 |
| PRSS1 | CELA3A | GABRB1 | CPA1 | CCL3 | SPP2 |
| SPP2 | REN | CPA2 | PTF1A | WNT3A | PTF1A |
| LEP | CALB1 | CALB1 | WNT3A | PTF1A | CPA1 |
| SERPINE1 | CELA1 | PTF1A | AGXT | ASCL1 | PLA2G1B |
| REN | PRSS3P2 | WNT3A | CCL3 | MBOAT4 | NTRK2 |
| GABRG1 | PRSS1 | SERPINA7 | SERPINA7 | AGXT | C9 |

Supplementary table 3 Full names and functions in the hub genes.

| N | Gene  symbol | Full name | Function |
| --- | --- | --- | --- |
| 1 | HRG | Histidine Rich Glycoprotein | This histidine-rich glycoprotein contains two cystatin-like domains and is located in plasma and platelets, It can inhibit rosette formation and interacts with heparin, thrombospondin and plasminogen. |
| 2 | AGXT | Alanine--Glyoxylate and Serine--Pyruvate Aminotransferase | Peroxisomal aminotransferase that catalyzes the transamination of glyoxylate to glycine and contributes to the glyoxylate detoxification |
| 3 | F2 | Coagulation Factor II, Thrombin | Thrombin, which cleaves bonds after Arg and Lys, converts fibrinogen to fibrin and activates factors V, VII, VIII, XIII, and, in complex with thrombomodulin, protein C |
| 4 | SERPINC1 | Serpin Family C Member 1 | Most important serine protease inhibitor in plasma that regulates the blood coagulation cascade. AT-III inhibits thrombin, matriptase-3/TMPRSS7, as well as factors IXa, Xa and XIa. |
| 5 | AHSG | Alpha 2-HS Glycoprotein | Promotes endocytosis, possesses opsonic properties and influences the mineral phase of bone. Shows affinity for calcium and barium ions. |
| 6 | REN | Renin | Renin is a highly specific endopeptidase, whose only known function is to generate angiotensin I from angiotensinogen in the plasma, initiating a cascade of reactions that produce an elevation of blood pressure and increased sodium retention by the kidney |
| 7 | APOC3 | Apolipoprotein C3 | Component of triglyceride-rich very low density lipoproteins (VLDL) and high density lipoproteins (HDL) in plasma. Plays a multifaceted role in triglyceride homeostasis (PubMed:18201179, PubMed:22510806). Intracellularly, promotes hepatic very low density lipoprotein 1 (VLDL1) assembly and secretion; extracellularly, attenuates hydrolysis and clearance of triglyceride-rich lipoproteins (TRLs) |
| 8 | HGFAC | Hepatocyte Growth Factor Activator | Activates hepatocyte growth factor (HGF) by converting it from a single chain to a heterodimeric form |
| 9 | SERPINE1 | Serpin Family E Member 1 | Is a primary inhibitor of tissue-type plasminogen activator (PLAT) and urokinase-type plasminogen activator (PLAU). As PLAT inhibitor, it is required for fibrinolysis down-regulation and is responsible for the controlled degradation of blood clots |
| 10 | LEP | Leptin | Key player in the regulation of energy balance and body weight control. Once released into the circulation, has central and peripheral effects by binding LEPR, found in many tissues, which results in the activation of several major signaling pathways |
| 11 | ADIPOQ | Adiponectin, C1Q And Collagen Domain Containing | Important adipokine involved in the control of fat metabolism and insulin sensitivity, with direct anti-diabetic, anti-atherogenic and anti-inflammatory activities. Stimulates AMPK phosphorylation and activation in the liver and the skeletal muscle, enhancing glucose utilization and fatty-acid combustion. |

Supplementary table 4 The impact of DNA methylation at the SERPINE1 CpG sites on the prognosis of PDAC.

| Name | Cancer | HR | CI | P.value | UCSC_RefGene_Group | Relation_to_UCSC_CpG_Island |
| --- | --- | --- | --- | --- | --- | --- |
| cg11353706 | PAAD | 2.103 | (1.252;3.533) | 0.004968 | Body | Open_Sea |
| cg17968347 | PAAD | 1.9 | (1.123;3.213) | 0.01668 | Body | Open_Sea |
| cg01975495 | PAAD | 0.551 | (0.329;0.921) | 0.022992 | Body | Open_Sea |
| cg15874872 | PAAD | 0.642 | (0.43;0.958) | 0.02999 | 5'UTR;1stExon | Open_Sea |
| cg08053846 | PAAD | 0.575 | (0.336;0.983) | 0.043153 | TSS1500 | Open_Sea |
| cg19722814 | PAAD | 0.597 | (0.36;0.99) | 0.045809 | TSS1500 | Open_Sea |
| cg25826546 | PAAD | 0.607 | (0.368;1.001) | 0.050462 | TSS1500 | Open_Sea |
| cg20438404 | PAAD | 0.673 | (0.421;1.077) | 0.099018 | TSS200 | Open_Sea |
| cg24539923 | PAAD | 0.75 | (0.502;1.121) | 0.16105 | TSS1500 | Open_Sea |
| cg08792542 | PAAD | 1.325 | (0.845;2.077) | 0.22038 | Body | Open_Sea |
| cg02273392 | PAAD | 0.79 | (0.531;1.176) | 0.245022 | 5'UTR;1stExon | Open_Sea |
| cg20583316 | PAAD | 0.795 | (0.533;1.188) | 0.263133 | 5'UTR;1stExon | Open_Sea |
| cg16617872 | PAAD | 1.222 | (0.818;1.824) | 0.326875 | Body | Open_Sea |
| cg12584355 | PAAD | 1.202 | (0.767;1.885) | 0.422094 | Body | Open_Sea |
| cg02704552 | PAAD | 0.908 | (0.606;1.358) | 0.637103 | 3'UTR | Open_Sea |
