# Supplementary figures and images for: Integrated multi-omics and single-cell analysis identify SERPINE1 as a key mediator of the inflammatory tumor microenvironment in PDAC
Source: Front Immunol. 2026 Jan 12;16:1716878. doi: 10.3389/fimmu.2025.1716878 (PMC12833254; doi:10.3389/fimmu.2025.1716878)

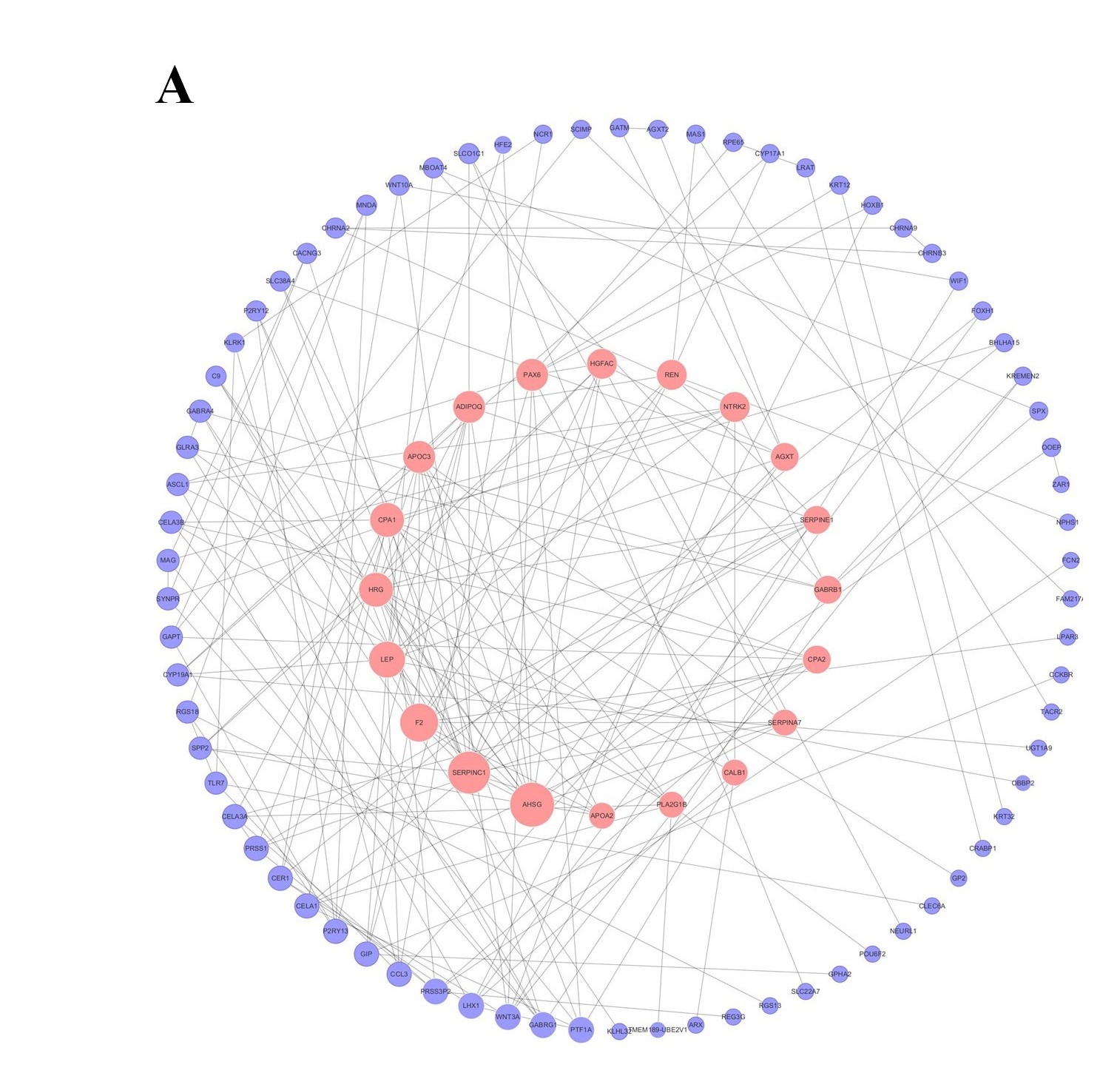

Supplement: Supplementary file 1 [file DataSheet1.zip › Supplementary Material/Supplementary Figure 1.jpg]

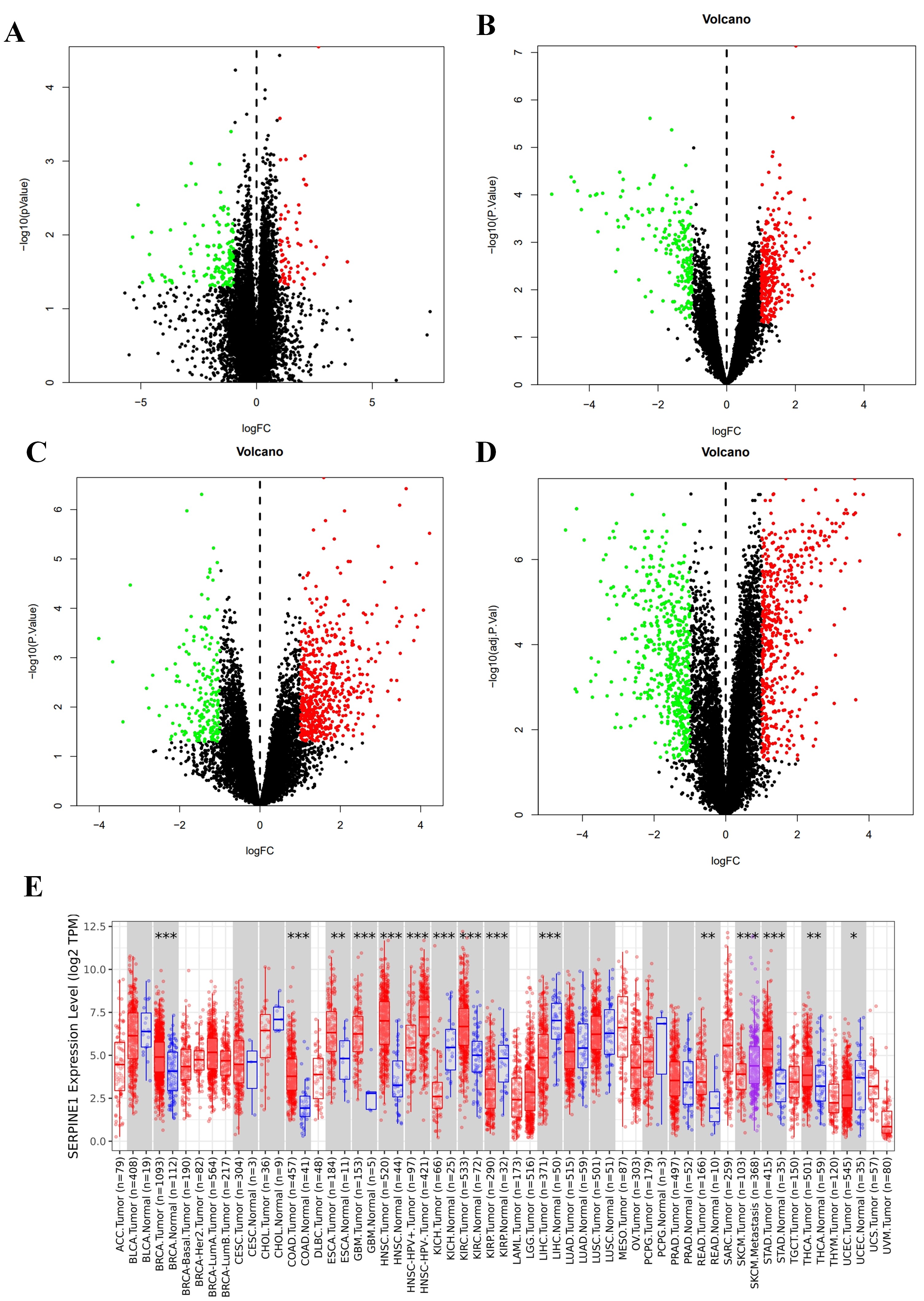

Supplement: Supplementary file 1 [file DataSheet1.zip › Supplementary Material/Supplementary Figure 2.jpg]

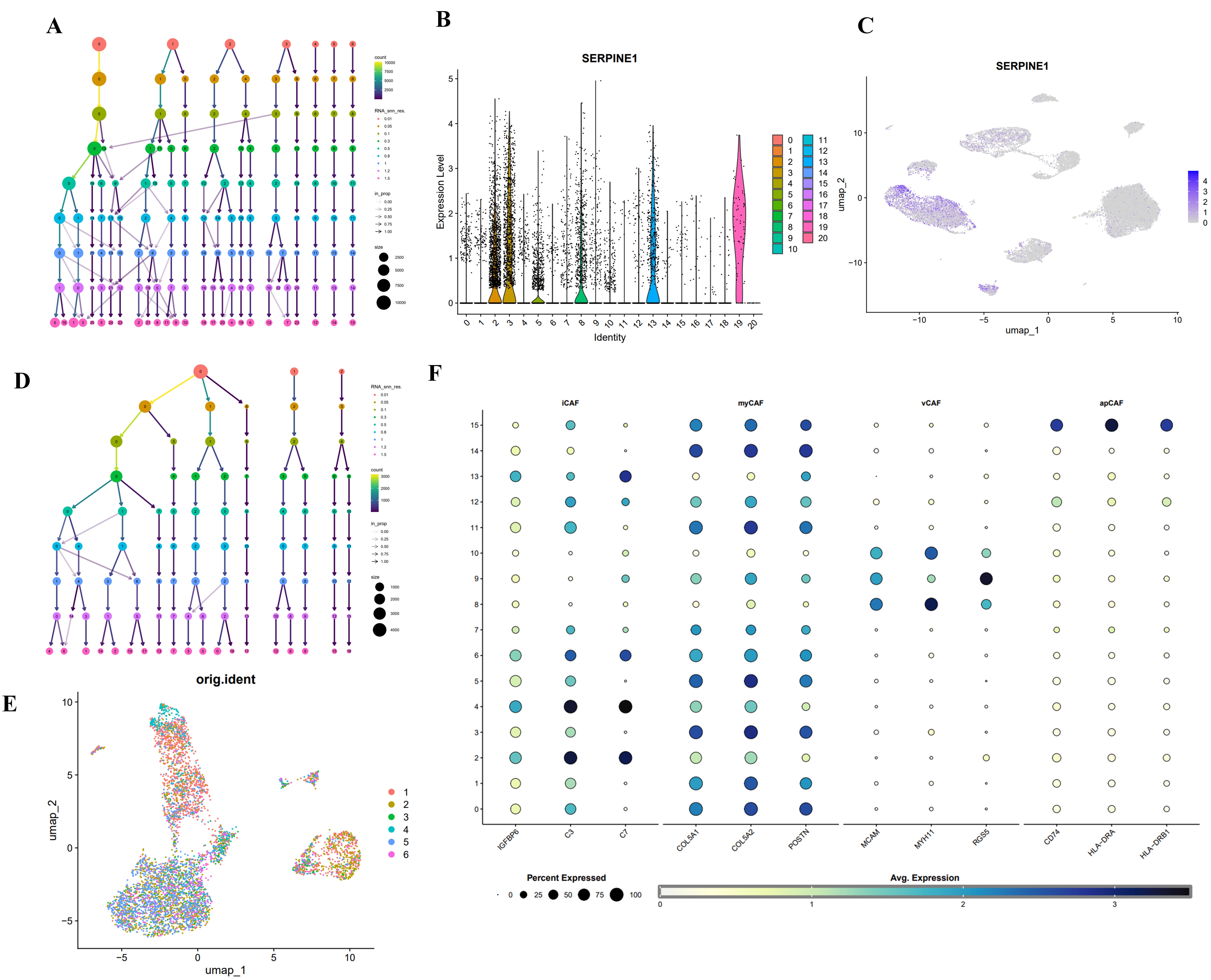

Supplement: Supplementary file 1 [file DataSheet1.zip › Supplementary Material/Supplementary Figure 4.jpg]

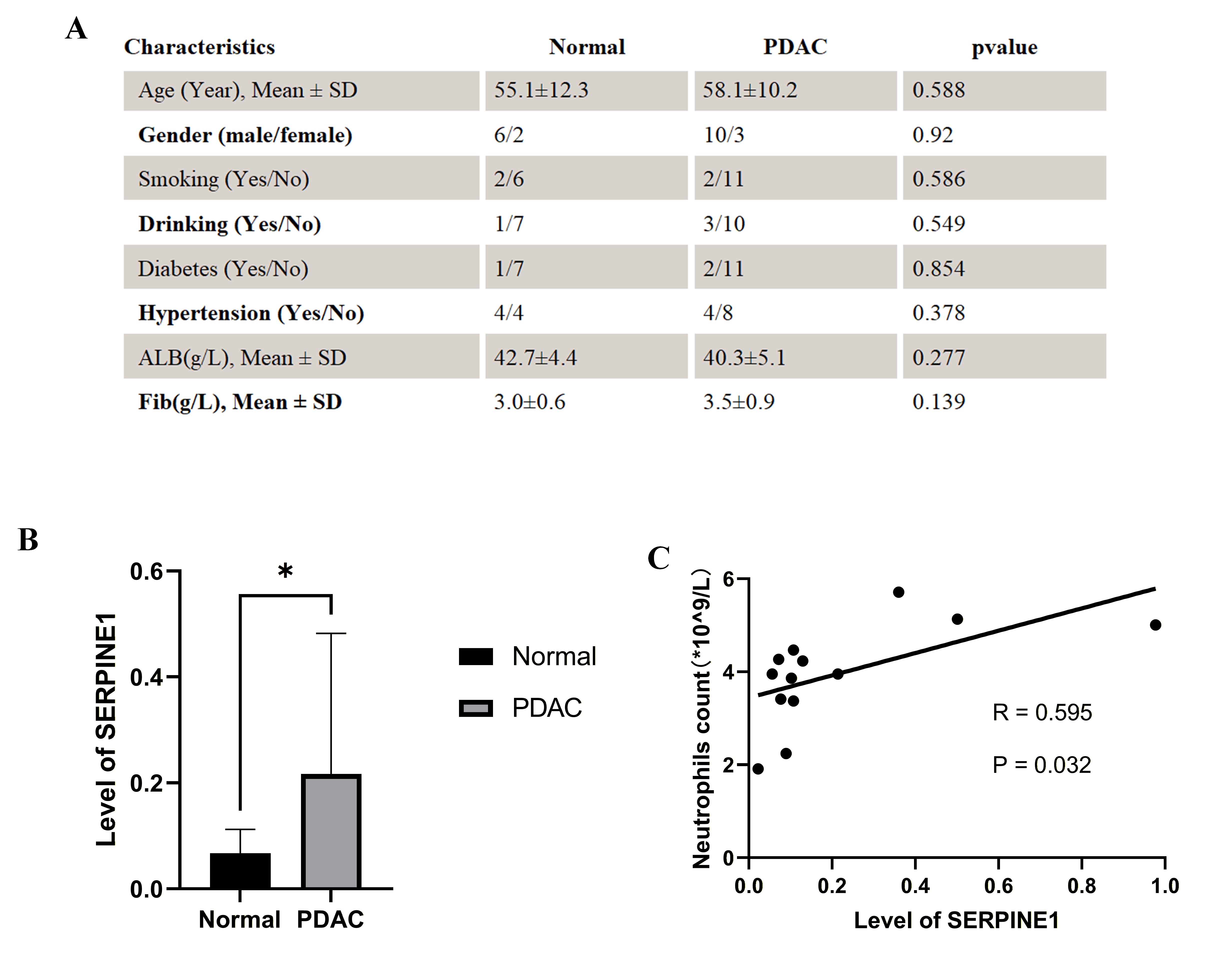

Supplement: Supplementary file 1 [file DataSheet1.zip › Supplementary Material/Supplementary Figure 5.jpg]

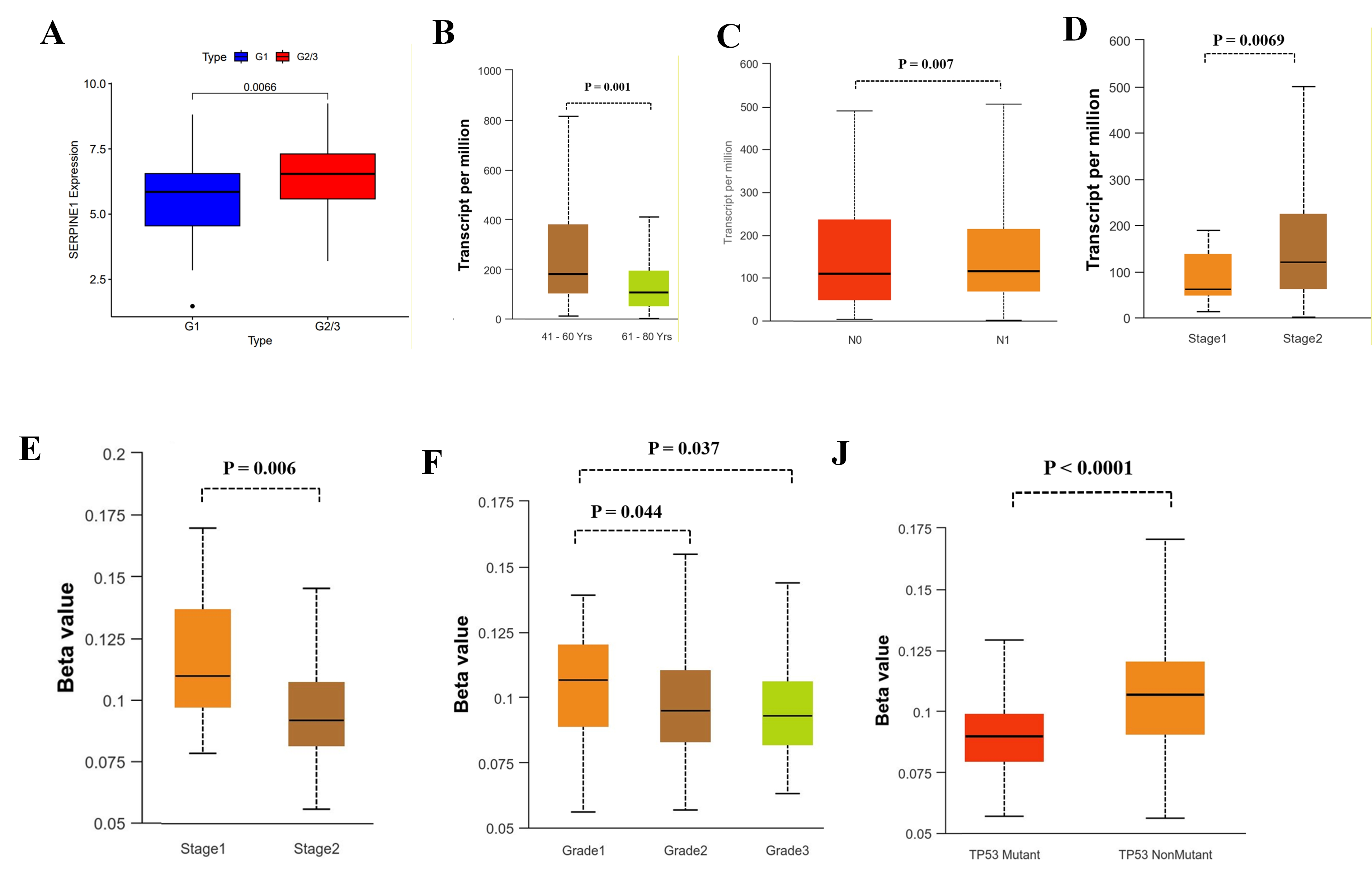

Supplement: Supplementary file 1 [file DataSheet1.zip › Supplementary Material/Supplementary Figure3.jpg]
